# PK-PD Modeling and Optimal Dosing Regimen of Acetylkitasamycin against *Streptococcus suis* in Piglets

**DOI:** 10.3390/antibiotics11020283

**Published:** 2022-02-21

**Authors:** Anxiong Huang, Feng Mao, Lingli Huang, Shuyu Xie, Yuanhu Pan, Wei Qu, Guyue Cheng, Zhenli Liu, Zonghui Yuan, Dapeng Peng, Haihong Hao

**Affiliations:** 1 National Reference Laboratory of Veterinary Drug Residues (HZAU) and MOA Key Laboratory for Detection of Veterinary Drug Residues, Wuhan 430070, China; anxionghuang@webmail.hzau.edu.cn (A.H.); maof890518@126.com (F.M.); huanglingli@mail.hzau.edu.cn (L.H.); snxsy1@126.com (S.X.); panyuanhu@mail.hzau.edu.cn (Y.P.); qw@mail.hzau.edu.cn (W.Q.); chengguyue@mail.hzau.edu.cn (G.C.); liuzhli009@mail.hzau.edu.cn (Z.L.); yuan5802@mail.hzau.edu.cn (Z.Y.); 2 MOA Laboratory for Risk Assessment of Quality and Safety of Livestock and Poultry Products, Wuhan 430070, China

**Keywords:** *Streptococcus suis*, acetylkitasamycin, PK-PD, dosing regimen, PELF

## Abstract

*Streptococcus suis* (*S. suis*) causes severe respiratory diseases in pigs and is also an important pathogen causing hidden dangers to public health and safety. Acetylkitasamycin is a new macrolide agent that has shown good activity to Gram-positive cocci such as *Streptococcus.* The purpose of this study was to perform pharmacokinetic–pharmacodynamic (PK-PD) modeling to formulate a dosing regimen of acetylkitasamycin for treatment of *S. suis* and to decrease the emergence of acetylkitasamycin-resistant *S. suis*. The minimal inhibitory concentration (MIC) of 110 *S. suis* isolates was determined by broth micro dilution method. The MIC_50_ of the 55 sensitive *S. suis* isolates was 1.21 μg/mL. The strain HB1607 with MIC close to MIC_50_ and high pathogenicity was used for the PK-PD experiments. The MIC and MBC of HB1607 in both MH broth and pulmonary epithelial lining fluid (PELF) was 1 and 2 μg/mL, respectively. The liquid chromatography–tandem mass spectrometry (LC-MS/MS) method was used to determine the concentration change of acetylkitasamycin in piglet plasma and PELF after intragastric administration of a single dose of 50 mg/kg b.w. acetylkitasamycin. The PK parameters were calculated by WinNolin software. The PK data showed that the maximum concentration (C_max_), peak time (T_max_), and area under the concentration–time curve (AUC) were 9.84 ± 0.39 μg/mL, 4.27 ± 0.19 h and 248.58 ± 21.17 h·μg/mL, respectively. Integration of the in vivo PK data and ex vivo PD data, an inhibition sigmoid E_max_ equation was established. The dosing regimen of acetylkitasamycin for the treatment *S. suis* infection established as 33.12 mg/kg b.w. every 12 h for 3 days. This study provided a reasonable dosing regimen for a new drug used in clinical treatment, which can effectively be used to treat *S. suis* infection and slow down the generation of drug resistance.

## 1. Introduction

*Streptococcus suis* (*S. suis*) is an important pathogen in swine, and can cause serious respiratory disease, resulting in great economic losses to the swine industry worldwide each year [1]. As a zoonotic pathogen *S. suis* can even cause human death [2]. Every year, a large number of antibiotics, including macrolides, are used for the treatment of respiratory disease. However, drug resistance has emerged as a result of the unreasonable use of drugs [3].

Macrolides are widely used in veterinary medicine to prevent and treat respiratory diseases and necrotic enteritis [4]. Acetylkitasamycin is a product of kitasamycin acetylation [5]. It has a similar spectrum of antibiotic activity to kitasamycin, but has superior pharmacokinetic (PK) properties and better palatability. Acetylkitamycin mainly has good antibacterial effect on Gram-positive cocci (e.g., *Streptococcus* and *Staphylococcus aureus*) and *mycoplasma* [6,7]. Many macrolides can accumulate in the lungs in order to achieve higher drug concentrations [8,9], so if acetylkitamycin can achieve higher concentrations than plasma, it would be suitable for the treatment of lung infections. The pharmacokinetics of acetylkitamycin in porcine ileum content has been revealed [10]; however, its PK in the respiratory tract remains largely unknown.

To reduce the occurrence of drug resistance, a reasonable dosing regimen is necessary. Pharmacokinetic–pharmacodynamic (PK-PD) properties are very important in the determination of dosing regimens of drugs [11,12]. Three parameters, including T > MIC, AUC/MIC and C_max_/MIC, are commonly used in PK-PD modeling [13]. T > MIC is used for time-dependent drugs, such as β-lactam antibiotics [14]; AUC/MIC is used for time-dependent drugs with significant PAE, such as glycopeptides and macrolides [15,16]; and AUC/MIC or C_max_/MIC are used for concentration-dependent drugs, such as aminoglycosides and fluoroquinolones [17,18].

Bronchoalveolar lavage is widely used for collecting samples from the respiratory tract [19], and it is also commonly used to collect pulmonary epithelial lining fluid (PELF) to study the PK of drugs in pigs [20]. The bronchoalveolar lavage has a large advantage in taking pulmonary samples, as compared to homogenized lung tissue [21], cotton swab [22], microdialysis [23], imaging techniques, and other methods [24].

When a new drug is used in clinical treatment, a reasonable dosing regimen is necessary. A reasonable dosing regimen can not only achieve the maximum therapeutic effect in clinical treatment, it can also slow down the occurrence of drug resistance and prolong the effective time of the drug. In this study, on the basis of a combined PK-PD study of acetylkitasamycin against *S. suis*, a reasonable dosing regimen of acetylkitasamycin for the treatment respiratory infection caused by *S. suis* was formulated to provide medication guidance for controlling clinical *S. suis* infection.

## 2. Materials and Methods

### 2.1. Drug and Reagents

Acetylkitasamycin was provided by Hai Na Chuan (a pharmaceutical company in Guangdong, China), and ahs five main components: A_6_A_7_, A_5_′, A_4_A_5_, A_1_A_3_, A_13_. A single product of acetylkitasamycin (A_6_A_7_, A_5_′, A_4_A_5_, A_1_A_3_, A_13_) was isolated and prepared by the National Veterinary Drug Residue Reference Laboratory of Huazhong Agricultural University, and the purity of all products was ≥90%. Acetonitrile, formic acid, and methanol were purchased from TEDIA (Fairfield, OH, USA). Normal hexane and ethyl acetate were purchased from Sinopharm Chemical Reagent Co., Ltd. (Beijing, China). In this experiment, all chemicals used were of analytical grade or higher. All water used was de-ionized water (Milli-Q Millipore Corp, Bedford, MA, USA).

### 2.2. Animals

Six weaned binary hybrid castrated healthy piglets weighing 20 ± 2 kg were purchased from Huazhong Agricultural University pig breeding farm. All the piglets were kept in the optimal environment. The piglets were fasted for 12 h before the experiments. All the animal experiments were approved by the Animal Ethics Committee of Huazhong Agricultural University (HZAUSW 2015-016) and the Animal Care Center, Hubei Science and Technology Agency in China (SYXK 2013-0044). All efforts were made to reduce the pain and adverse effects of the animals.

### 2.3. PD Study of Acetylkitasamycin against S. suis

#### 2.3.1. Isolation and Identification of *S. suis*

From the year 2013 to 2015, 110 *S. suis* were isolated from pig farms in Hubei, Henan, Guangdong, Hebei, Jiangsu and Shandong provinces in China. The respiratory tract samples of pigs with respiratory diseases were collected, then inoculated into Tryptic Soy Agar (TSA) medium supplemented with 5% fetal bovine serum. After culturing for 18–24 h under appropriate conditions, suspicious colonies were picked out for PCR identification. The primers required for PCR identification are designed based on the nucleotide sequence GDH of the specific gene of *S. suis* [25].

#### 2.3.2. Determination of MIC, MBC, MPC and PAE

The minimal inhibitory concentration (MIC) of acetylkitasamycin and its five main components against *S. suis* strains were determined in MH broth and PELF by broth micro dilution method according to the CLSI 2007 [26,27]. The *S. suis* ATCC 49619 and *E. coli* ATCC 25922 strains were used as the quality control strain for antibiotic susceptibility determination. According to the MIC_50_ values of sensitive strains, an *S. suis* HB1607 strain with its MIC similar with MIC_50_, was used for PD study of acetylkitasamycin.

The supernatant was sucked up from the MIC determination wells to the TSA in order to determine the minimal bactericidal concentration (MBC) [28]. The agar dilution method was used to determine the mutant prevention concentration (MPC) of acetylkitasamycin [28]. The 10^10^ CFU/mL bacterial were inoculated on the agar plates containing continuous concentrations of acetylkitasamycin (1MIC, 2MIC, 4MIC, 8MIC, 16MIC, 32MIC) and cultured at 37 °C for 72 h, and the lowest concentration without bacterial growth was MPC.

Post-antibiotic effect (PAE) was estimated by incubating bacteria with drug for a period of time and then removal of drug [29]. First, the bacteria were incubated with 1MIC, 2MIC, 4MIC of drugs for 1 and 2 h; second, the drugs were removed by washing with new medium; third, 100 μL new incubation was sucked up at different time points and counted by plating on TSA. Then the recovery growth kinetic curves were established for computing the PAE. The PAE was calculated as follows: PAE = T − C, where T is the time required for viable counts of bacteria to increase by 1 − log10 CFU in drug removal phase, respectively; C is the time for untreated control.

#### 2.3.3. In Vitro and Ex Vivo Bacterial Killing Curves

Prepare TSB (Tryptic Soy Broth with 5% fetal bovine serum) containing different concentrations of acetylkitasamycin (1/2MIC, 1MIC, 2MIC, 4MIC, 8MIC, 16MIC, 32MIC) for in vitro bacteria killing curves [30]. The different concentrations of drugs and bacteria (10^6^ CFU/mL) were incubated at 37 °C, 5% CO_2_. At each of the time points (0, 1, 2, 4, 6, 8, 12, 24 h), 100 µL of medium was sucked up, diluted with saline and coating on TSA, and the colony forming unit (CFU) changes after incubation in a 37 °C, 5% CO_2_ environment for 24 h were counted.

The ex vivo time-killing curves were estimated in PELF samples taken from piglets at different time points (0, 0.5, 1, 2, 4, 6, 8, 10, 12, 24, 36, 48, 72 and 96 h) after intragastric administration with 50 mg/kg b.w. acetylkitasamycin [30]. The method was the same as that in an earlier in vitro study. Each concentration test was performed in triplicate.

### 2.4. PK Study of Acetylkitasamycin in Piglets

#### 2.4.1. Animal Experiment and Sample Collection for PK Study

Acetylkitasamycin was administrated in six piglets with a single dose of 50 mg/kg b.w. by intragastric administration. After administration, 5 mL blood samples were collected through the anterior vena cava at 0.5, 1, 2, 4, 6, 8, 12, 24, 36, 48, 72 and 96 h. Plasma was separated from blood by centrifugation at 3500 rpm for 10 min.

To collect PELF samples, atropine (0.05 mg/kg) and propofol (9~15 mg/kg) were given intramuscularly and intravenously 30 min for anesthesia. Standardized Bronchoaveolar Lavage was performed as previously described [31,32], with an electronic fiber opticbron choscope (Kangmei GU-180 VET, Zhuhai, China) inserted in the right middle lung lobe. A 50 mL volume of normal saline was instilled in the lobe, and was aspirated into a 50 mL centrifugal tube. The PELF samples were collected at 0, 0.5, 1, 2, 4, 6, 8, 10, 12, 24, 36, 48, 72 and 96 h, and centrifuged at 800 rpm for 10 min.

#### 2.4.2. Assay of Acetylkitasamycin and Kitasamycin Every Component in Plasma and PELF

The urea dilution method was used to determine the volume of PELF, as described previously [33]. To determine the urea concentration in lavage fluid samples, a biochemical analyzer machine (SYNCHRON CX4 PRO) was used. Estimation of the volume of PELF was done by the urea dilution method. The final concentration of acetylkitasamycin in PELF (C_PELF_) was derived from the following equation: $C_{\mathrm{PELF}}=C_{\mathrm{BAL}}\times \left(\frac{{\mathrm{Urea}}_{\mathrm{PLASMA}}}{{\mathrm{Urea}}_{\mathrm{PELF}}}\right)$.

Quantitation of acetylkitasamycin and kitasamycin in every component of piglet plasma and PELF was conducted using the sensitive and selective high-performance liquid chromatographic mass spectrometry (LC-MS/MS) method [34]. The plasma specimens (0.25 mL) and BAL specimens (0.25 mL) were thawed and added to a 10 mL centrifuge tube with 0.75 mL acetonitrile, and vortexed for 3 min (12,000 r/10 min); 1 mL ethylacetate was added to the supernatant and vortexed for 3 min (12,000 r/10 min), and then the supernatant was taken out. The above operation process was repeated. The two supernatants were merged and blown dry with nitrogen. Then the residue was reconstituted in 0.25 mL solution (0.1% Formic acid water (60): Acetonitrile (40)).

All PK parameters of plasma and PELF were performed using WinNonlin software (version 5.2.1, Pharsight Corporation, Mountain View, CA, USA). Each piglet’s drug concentrations were depicted on semilogarithmic graphs to choose appropriate PK compartmental models.

Taking into account that acetylkitasamycin has several components, the weight coefficient was introduced in this study. Weight coefficient (w_j_) can be customized for each component AUC_0-∞__j_ in consideration of the total AUC_0-∞__t_ ratio. Every monomer composition of acetylkitasamycin in the concentration of PELF was given their own weight coefficient, and then the total concentration was calculated (C_T_).

$$w_{j}=\frac{{\mathrm{AUC}}_{0-\infty j}}{{\mathrm{AUC}}_{0-\infty t}}\left(j=A_{6}A_{7},{\;A}_{5}^{\prime },A_{4}A_{5},A_{1}A_{3},{\;A}_{13}\right)$$

$${\mathrm{AUC}}_{0-\infty t}={\mathrm{AUC}}_{0-\infty A6A7}+{\mathrm{AUC}}_{0-\infty {A_{5}}^{\prime }}+{\mathrm{AUC}}_{0-\infty A4A5}+{\mathrm{AUC}}_{0-\infty A1A3}+{\mathrm{AUC}}_{0-\infty A13}$$

$$C_{T}=w_{A6A7}\times C_{A6A7}+w_{{A_{5}}^{\prime }}\times C_{{A_{5}}^{\prime }}+w_{A4A5}\times C_{A4A5}+w_{A1A3}\times C_{A1A3}+w_{A13}\times C_{A13}$$

### 2.5. PK-PD Integration and Modeling

All the parameters were calculated using WinNonlin 5.2 software. The surrogate parameters (C_max_/MIC, AUC_24h_/MIC, T > MIC) of PELF were determined after intragastric administration of acetylkitasamycin, used for in established vitro MIC and in vivo PK relationships.

The inhibitory sigmoid E_max_ model was used to establish the relationship between ex vivo AUC_24h_/MIC ratio and the bacteria decrease in the PELF of piglets [16]. The model formula can be described as follows: $\;E=E_{0}-\frac{{\mathrm{PD}}_{\max}\cdot C^{N}}{C^{N}+{\mathrm{EC}}_{50}^{N}}$. E is the summary PD endpoint, and E_0_ is the effect representing the value of the PD endpoint without drug treatment (i.e., the value of the summary endpoint when the PK-PD index is 0). C is one of the three PK-PD indices, as defined above, and PD_max_ is the maximum effect (in relation to E_0_) indicated by the plateau where further exposure does not result in further killing. EC_50_ is the magnitude of C that is needed to achieve 50% of E_max_ − E_0_, and N is the sigmoidicity factor. The PD target under different efficiencies (E = 0, −3 and −4 (bacteriostasis, bactericidal and eradication)) was determined using the Sigmoid E_max_ equation [35].

### 2.6. Dosage Designation

Daily dose was calculated using the dosage equation: $\mathrm{Dose}=\frac{\mathrm{CL}\times \left({\mathrm{AUC}}_{24h}/\mathrm{MIC}\right)\times \mathrm{MIC}}{F\times f_{u}}$, where CL is the clearance, AUC_24h_/MIC is the targeted endpoint for optimal efficacy, f_u_ is free fraction of drug in plasma, F is the bioavailability factor(from 0 to 1) [36]. The f_u_ in epithelial lining fluid can be ignored, because of the low albumin levels in epithelial lining fluid [33].

To investigate the effect of different dosing regimens, the PD model indicates that the bacterial growth rate in the function of acetylkitasamycin concentration is combined with PK model, and simulations were performed with MlxPlore software (version-1.1.1, Lixoft, Orsay, France).

## 3. Results

### 3.1. PD Study of Acetylkitasamycin on S. suis

#### 3.1.1. MIC of Acetylkitasamycin against *S. suis* Isolates

**Figure 1:**
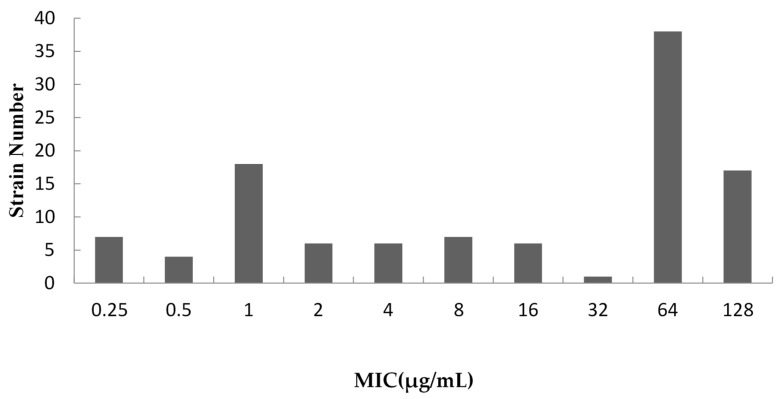
Acetylkitasamycin MIC distribution of 110 *S. suis* strains isolated.

All the MIC of acetylkitasamycin against 110 *S. suis* were in the range of 0.25~128 μg/mL. MIC distribution of the acetylkitasamycin against 110 *S. suis* is shown in Figure 1. Non-linear least squares regression was used to fit a series distribution of log_2_-transformed MIC data to a range of symmetrical ‘bell-shaped’ theoretical population distributions which was conducted in GraphPad Prism 5 software (Table 1 and Table 2). The results manifested the smallest difference between the estimated and true number of isolates in the subset of 16 μg/mL (Table 2), so 16 μg/mL was set as the wild-type cutoff value. To select a sensitive strain, the wild-type cutoff value was set as interpretive criteria. The MIC_50_ and MIC_90_ of 110 strains were 9.10 μg/mL and 100.31 μg/mL, respectively. According to the interpretation criteria, which were set as described above, a total of 55 strains were sensitive strains, and the MIC_50_ and MIC_90_ were 1.21 μg/mL and 6.94 μg/mL, respectively (Table 3).

#### 3.1.2. MIC, MBC, MPC and PAE of Acetylkitasamycin against *S. suis* HB1607

Through the toxicity experiment in mice the strain number HB1607 with serotype 2 which MIC close to MIC_50_ was used to study the antimicrobial activity of acetylkitasamycin in vitro and ex vivo.

MICs of acetylkitasamycin in MHB and PELF against *S. suis* HB1607 were 1 μg/mL, MBC were 2 μg/mL and 4 μg/mL, respectively. The MICs of single components of acetylkitasamycin against *S. suis* HB1607 in MHB were both 1 μg/mL, and MBCs were both 2 μg/mL. In addition, the MICs of metabolites kitasamycin against *S. suis* HB1607 were both 2 μg/mL, MBCs were both 8 μg/mL. The MPC of acetylkitasamycin against *S. suis* HB1607 in MHB was 5 μg/mL. The PAE of acetylkitasamycin against *S. suis* HB1607 is shown in Table 4.

#### 3.1.3. In Vitro and Ex Vivo Antimicrobial Activity

**Figure 2:**
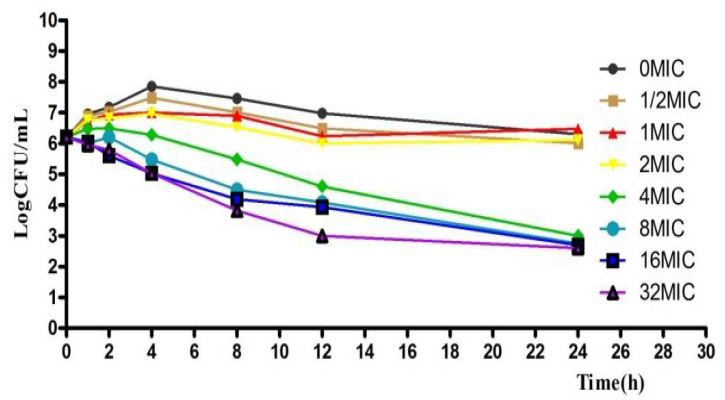
In vitro antibacterial of acetylkitasamycin against *S. suis* in MHB.

According to the MIC values, a series of concentrations of acetylkitasamycin was prepared to describe the killing curve. The curves were characteristically time dependent with significant PAE (Figure 2). Along with the extended time, the bacteria number decreased slowly, but the bactericidal activity was enhanced. In addition, when exposed to the higher concentrations (≥1 μg/mL) of acetylkitasamycin for 4 h, the bacteria decreased, but not to an undetectable level (<30 CFU).

**Figure 3:**
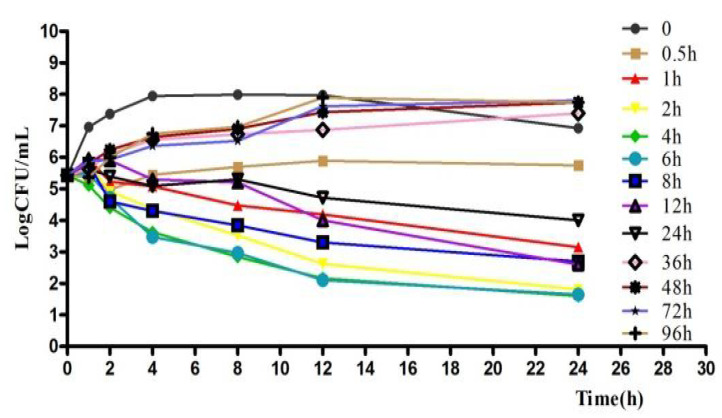
Ex vivo antibacterial activity of acetylkitasamycin in PELF of piglets against *S. suis* after intragastric administration.

The ex vivo killing curve results showed that acetylkitasamycin was also time-dependent (Figure 3), and was consistent with the in vitro killing curve. Along with time prolongation and the increase in concentration, the number of bacteria decreased sharply.

### 3.2. PK Study of Acetylkitasamycin in Piglets

The concentrations of total and every component of acetylkitasamycin in plasma and PELF were best fit first order two compartment model and the PK parameters were calculated by WinNonlin 5.2 (Table 5). After intragastric administration, the concentrations of total and every component of acetylkitasamycin in plasma were below the 1 μg/mL, and only A_5_′ and A_4_A_5_ were detected in plasma with continuous concentrations. C_maxPELF_/C_maxplasma_ of A_5_′ was 31.54, AUC_PELF_/AUC_plasma_ was 141.04; C_maxPELF_/C_maxplasma_ of A_4_A_5_ was 44.38, AUC_PELF_/AUC_plasma_ was 241.65. In addition, the AUC, C_max_, T_max_ in PELF were 248.58 h·μg/mL, 9.85 μg/mL and 4.27 h, respectively.

**Figure 4:**
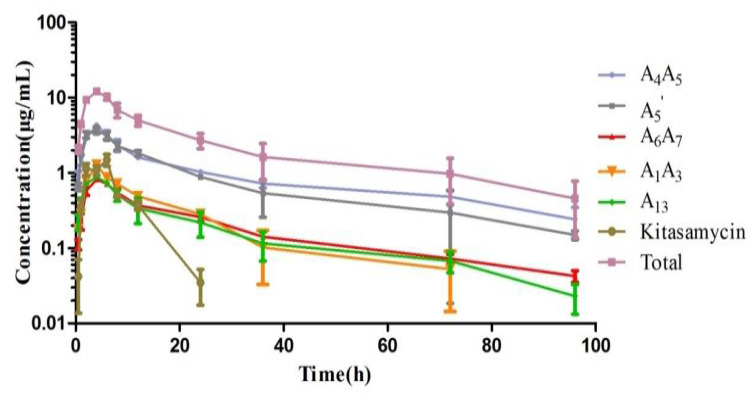
Acetylkitasamycin concentrations in PELF-versus-time curves plotted semilogarthmically for data obtained after intragastric administration.

The concentrations of every component of acetylkitasamycin and its main metabolite kitasamycin in PELF were determined by LC-MS/MS after intragastric administration is shown in Figure 4.

### 3.3. PK-PD Model Integration

The PK-PD parameters AUC_24h_/MIC, AUC_24h_/MPC, and T > MIC, T > MPC, integrating the PK-PD of acetylkitasamycin against *S. suis*, were 139.54 ± 5.30 h, 27.91 ± 1.06 h, and 45.54 h, 11.08 h, respectively. The inhibitory sigmoid E_max_ model flawless expressed the relationship between antimicrobial efficacy of acetylkitasamycin and the PK-PD parameter of AUC_24h_/MIC ratio in PELF (Table 6). The parameters acquired were the values of N, E_0_, PD_max_, EC_50_ and AUC_24h_/MIC, which represent different levels of antibacterial activity (Table 6).

### 3.4. Estimation and Assessment of Dose

According to the dosage equation, the optimal dose was calculated. CL/F was 202.49 ± 15.69 mL/h/kg, calculated by WinNonlin s oftware. The MIC_50_ was 1 μg/mL, and the fu was ignored. When E = 0 (bacteriostatic action), the AUC_24h_/MIC was 57.65 h, the dosage calculated for bacteriostatic was 11.67 mg/kg. When E = −3 (bactericidal action), the AUC_24h_/MIC was 163.56 h, the dosage calculated for bactericidal was 33.12 mg/kg. When E = −4 (eradication action), the AUC_24h_/MIC was 407.12 h, the dosage calculated for eradication was 82.44 mg/kg.

**Figure 5:**
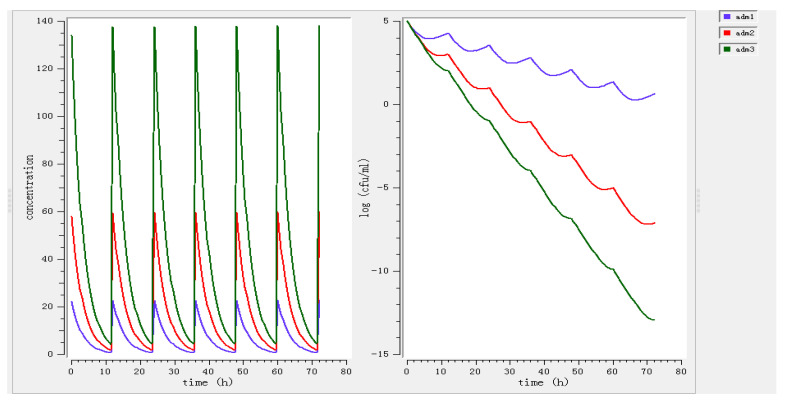
Model predictions of drug concentration (**left**) and bacterial growth (**right**) at different dose regimens with 12 h intervals by Mlxplore (adm1: Preventive dose, adm2: Therapeutic dose, adm3: Eradication dose).

MlxPlore software was used to simulate the effects of different doses (11.67, 33.12, 82.44 mg/kg) in vivo (Figure 5). On the basis of Figure 5, higher doses (33.12, 82.44 mg/kg) possessed bactericidal or eradication action during 0–12 h, but the bacterial regrowth occurred under the lower dose (11.67 mg/kg) treatment. Different dosing regimens (11.67 mg/kg every 12 h, 33.12 mg/kg every 12 h, 82.44 mg/kg every 12 h) were employed, simulating 3 days for treatment (Figure 5). At least 33.12 mg/kg every 12 h was sufficient to achieve bactericidal activity in PELF. Therefore, the dosing regimen of acetylkitasamycin for the treatment *S. suis* infection established as 33.12 mg/kg b.w. every 12 h for 3 days.

## 4. Discussion

There is a substantial lack of preclinical and clinical PK data for acetylkitasamycin, and only the PK study in the ileum content has been studied [10]. The previous results indicated that the concentrations of every component of acetylkitasamycin in plasma were much lower than the MIC (1 μg/mL). On the contrary, the concentrations in PELF (9.84 μg/mL) were more than 10 times that of plasma. This result is the same as that of other macrolides; the concentrations of azithromycin and clarithromycin in foal PELF were more than 10 times that in foal plasma [8].

Recent evidence from this experiment and other studies (including animals and folks) have highlighted the importance of drug concentrations at the infection site in predicting the appropriate dosage for therapy. In addition to the macrolide drugs, other drugs such as doxycycline [37], moxifloxacin [38], linezolid [39] also have a higher concentrations in PELF. Therefore, the PK-PD study using the drug concentrations at the infection site can better reflect the actual clinical administration situation than using the drug concentrations in the plasma [8].

The T_max_ values of the five main components A_6_A_7_, A_5_′, A_4_A_5_, A_1_A_3_ and A_13_ of acetylkitasamycin were 4.54 h, 3.81 h, 3.90 h, 4.34 h and 3.74 h, respectively. Analysis shows that there is no significant difference from the total T_max_ value 4.27 h. Meanwhile, there was a significant difference between C_max_ and AUC, due to the content of each component is different in pharmaceutical ingredients. Just like acetylkitasamycin, bitespiramycin has many components (Bitespiramycin I, II, III). After giving 80 mg/kg bitespiramycin to rats, the three main components reaching T_max_ in plasma were 2.37 h, 2.69 h and 2.84 h, respectively, with no significant difference. However, there were significant differences in C_max_ and AUC for the three main components [40].

To reveal the influence of every component in the total concentrations, weight coefficient (*wj*) was introduced [41]. Every monomer composition of acetylkitasamycin in PELF concentrations was given its own weight coefficient, in order to calculate the total concentration (*C_T_*). This was superior to simply summing the concentrations (enrofloxacin + ciprofloxacin) at the different time points [42]. Through the software WinNonlin 5.2, the values of C_max_, T_max_ and AUC were 9.84 μg/mL, 4.27 h and 248.58 h·μg/mL, respectively.

A very limited veterinary breakpoints has been properly established, and even most of testing laboratories continue routinely use human breakpoints [43]. Zafar’s research demonstrated that *S. suis* resistance to macrolides increased steeply from 2002 to 2009, from 13% to 29.7% [44]. It is necessary to establish the breakpoint of acetylkitasamycin against *S. suis*. In this study, the wild-type cutoff value, one of the three cutoff values necessary to establish a breakpoint, was established as 16 μg/mL. There were 55 *S. suis* strains, where MIC ≤ 16 μg/mL, and MIC_50_ and MIC_90_ were 1.21 μg/mL and 6.94 μg/mL, respectively.

The MIC of the clinical separation bacteria *S. suis* HB1607 in MHB was not significantly greater than in PELF. It is worth noting that the total protein concentration in MHB used in this research was 3.78 g/L [45], and the corresponding concentrations in PELF were 0.25~0.62 g/L [46]. Therefore, the drug in PELF were perceived to be in free form when it comes to protein binding rates in PELF [33].

The PD target of AUC_24h_/MIC ≥ 30 was an indicator for the success of therapy and preventing emergence of resistance of macrolide [47]. In this study, the AUC_24h_/MIC obtained for bactericidal action in PELF was 137.71, which was much larger than 30. This may be because the concentrations in PELF were much higher than that in plasma. Zhanel [48] simulated the effect of azithromycin against *S. pneumoniae* in vitro, and determined the PD target in serum, AUC_24h_/MIC ≥ 36.7, to be bactericidal. Schentag [49] believed that AUC_24h_/MIC was a good predictor of the effect of erythromycin in human plasma, with reported PD target AUC_24h_/MIC = 53. Furthermore, some studies in the literature show that the parameter of AUC/MIC indicates that the effect is better than T > MIC, such as macrolides, azalides, ketolides and clindamycin [17,50].

In the past, dosing regimens for clinically used antimicrobials were generally developed on the basis of related PK data obtained in in vitro measurements of antibacterial activity or the empirical clinical treatment, which are not based on solid PK-PD data. However, overuse and misuse of drugs is considered to be the primary factor that increases bacteria resistance in both humans and animals. PK-PD integration is regarded as a complimentary approach to PK-PD modeling for predicting the adequacy of the regimen in clinical subjects. According to the PK and PD data in this experiment, the dosages required to obtain different effects (bacteriostatic, bactericidal, eradication) were 11.67 mg/kg, 33.12 mg/kg, 82.44 mg/kg, respectively. After the professional software simulation, different dosages showed that 33.12 mg/ kg treatment every 12 h for 3 days was sufficient to cure *S. suis* (HB1607). Therefore, the dosing regimen of acetylkitasamycin to treat *S. suis* was established as 33.12 mg/kg b.w. every 12 h for 3 days.

## 5. Limitations

According to the MIC determination of acetylkitasamycin against 110 *S. suis* strains, HB1607 with MIC close to MIC_50_ and high pathogenicity was selected for the follow-up PD study, which was representative. However, considering the potential PD variability of each strain, this study cannot represent PD studies of acetylkitasamycin against all *S. suis*.

This study employed post-anesthesia sampling, a procedure necessary for animal welfare. Anesthesia may potentially affect the metabolism of drugs, but there are no reports of anesthetics affecting the metabolism of macrolides.

As shown in Table 5, there were large differences of drug concentrations in plasma and PELF, and only two components were detected in plasma with continuous concentrations. The authors believe that using plasma drug concentrations to calculate the administered dose would increase the dose of drug used during treatment and increase unnecessary risk. The purpose of this study was to determine the optimal dosing regimen of acetylkitasamycin for the treatment of *S. suis* infection, the PK data in target tissue will be more useful for the precise use of the drug to treatment the pneumonia. Although *S. suis* may cause systemic infections, the focus here was on pulmonary infections. Therefore, the drug concentrations in the PELF of lung tissue were used to calculate the dosing regimen.

## 6. Conclusions

The purpose of this study was to determine the adequacy regimen of acetylkitasamycin that could be effectively used to cure pigs infected with *S. suis*. The dosage determined was based on PK and PD data analysis. PD data were acquired from analysis of the the static time kill curves obtained from ex vivo experiment. The inhibition E_max_ equation was used to calculate the dosage. The dosing regimen of acetylkitasamycin to treat *S. suis* wasestablished as 33.12 mg/kg b.w. every 12 h for 3 days.

## Figures and Tables

**Table 1:** Distribution Log_2_MICs and cumulative distribution Log_2_MICs.

Parameter	Distribution Log_2_MICs									
	−2	−1	0	1	2	3	4	5	6	7
Counts	7	4	18	6	6	7	6	1	38	17
Cumulative	7	11	29	35	41	48	54	55	93	110

**Table 2:** Optimum non-linear least squares regression fitting of pooled MICs (μg/mL).

Subset Fitted	Number of Isolates						Mean MIC (Log_2_)				Standard Deviation (Log_2_)			
	True	Est	Diff	ASE	Est/ASE	95% CI	Est	ASE	Est/ASE	95% CI	Est	ASE	Est/ASE	95% CI
≤2	35	39	4	11.88	3.2	−118, 8190	−0.569	0.634	−0.9	−8.635, 7.495	1.171	0.668	2.6	0.0, 9.659
≤4	41	42	1	4.678	8.9	21.77, 62.03	−0.443	0.300	−1.5	−1.736, 0.8502	1.297	0.371	3.4	0.0, 2.896
≤8	48	47	−1	4.492	10.4	33.65, 62.24	−0.113	0.314	−0.4	−1.104, 0.8778	1.656	0.389	4.2	−1.104, 0.87
≤16 ^a^	54	54	0	4.549	11.8	41.35, 66.61	0.252	0.332	0.7	−0.671, 1.176	2.037	0.417	4.8	0.878, 3.195
≤32	55	56	1	2.964	18.8	47.54, 62.78	0.328	0.245	1.3	−0.303, 0.960	2.118	0.245	8.6	−0.303, 0.96

Note: Est, non-linear regression estimate of value; Diff, estimate of N minus true; ASE, asymptotic standard error; Est/ASE, estimate divided by asymptotic standard error; CI, Confidence interval. ^a^, this subset gave the smallest difference between the estimated and true number of isolates in the subset, and was therefore selected for estimates of the mean and standard deviation for the antibiotic–bacterium concentration.

**Table 3:** Susceptibilities of isolated *S. suis* strains.

Antibiotic	*S. suis* (110 strains) ^b^			*S. suis* (55 strains) ^c^		
Acetylkitasamycin (μg/mL)	MIC_50_	MIC_90_	Range	MIC_50_	MIC_90_	Range
	9.10	100.23	0.25–128	1.21	6.94	0.25–16

Note: ^b^, the total bacterial number; ^c^, the susceptible strain based on this paper set interpretive criteria.

**Table 4:** The PAE of acetylkitasamycin against *S. suis*.

Concentration (µg/mL)	Post-Antibiotic Effect (PAE)	
	Expose 1 h	Expose 2 h
1MIC	0.92	1.72
2MIC	1.59	2.33
4MIC	1.88	2.96

**Table 5:** PK of acetylkitasamycin and every component in plasma and PELF after intragastric administration (*n* = 6).

Parameter	Acetylkitasamycin (Plasma)		Acetylkitasamycin (PELF)					
	A_5_′	A_4_A_5_	A_6_A_7_	A_5_′	A_4_A_5_	A_1_A_3_	A_13_	Total
α (1/h)	0.56 ±0.04	0.46 ±0.02	0.25 ±0.01	0.3 ±0.01	0.28 ±0.02	0.25 ±0.02	0.3 ±0.02	0.27 ±0.03
β (1/h)	0.09 ±0.01	0.19 ±0.02	0.018 ±0.04	0.022 ±0.02	0.012 ±0.01	0.017 ±0.01	0.018 ±0.02	0.016 ±0.03
T_1/2_01 (h)	1.2 ±0.08	1.49 ±0.13	2.72 ±0.09	2.29 ±0.12	2.42 ±0.28	2.75 ±0.22	2.26 ±0.16	2.54 ±0.09
T_1/2_10 (h)	1.4 ±0.21	1.5 ±0.16	8.19 ±0.63	6.57 ±0.47	9.4 ±2.9	34.93 ±56.77	6.66 ±1.49	7.25 ±0.78
T_1/2_α (h)	1.25 ±0.13	1.5 ±0.16	2.75 ±0.11	2.31 ±0.13	2.42 ±0.25	2.75 ±0.23	2.28 ±0.17	2.62 ±0.27
T_1/2β_ (h)	7.47 ±0.54	3.56 ±0.25	39.52 ±3.27	32.16 ±3.17	63.89 ±5.58	42.7 ±4.32	53.12 ±4.83	46 ±4.28
AUC (h·μg/mL)	0.57 ±0.15	0.49 ±0.11	20.55 ±2.25	80.39 ±3.76	118.41 ±24.71	23.95 ±3.26	18.88 ±3.05	248.58 ±21.17
T_max_ (h)	1.8 ±0.08	2.15 ±0.11	4.54 ±0.07	3.81 ±0.14	3.9 ±0.33	4.34 ±0.35	3.74 ±0.36	4.27 ±0.19
C_max_ (μg/mL)	0.11 ±0.01	0.08 ±0.01	0.71 ±0.05	3.47 ±0.32	3.55 ±0.25	0.98 ±0.02	0.8 ±0.04	9.84 ±0.39
CL/F (mL/h/kg)	87.33 ±7.32	101.57 10.08±	2461.14 ±258.80	623.26 ±28.32	437.35 ±72.92	2123.08 ±266.31	2709.83 ±384.11	202.49 ±15.69
Vd/F (mL/kg)	82.49 ±3.22	0.37 ±0.04	73,189 ±7332	14,745 ±2561	23,567 ±2142	52,619 ±6252	111,655 ±5254	22,895 ±4637

Note: α and β: exponential coefficients; T_1/2_01: absorption rate constant; T_1/2_10: central compartment elimination rate constant; T_1/2α_: half-life of α phase; T_1/2β_: half-life of β phase; AUC: area under the curve of plasma concentration-time; T_max_: the time point of maximum plasma concentration of the drug; C_max_: the maximum plasma concentration; CL/F: the apparent volume of the central compartment cleared of drug per unit time; Vd/F: Apparent volume of distribution based on the terminal elimination phase.

**Table 6:** PK-PD integration parameters for acetylkitasamycin in PELF after intragastric administration at a dose of 50 mg/kg b.w. (*n* = 6).

Time (h)	C_vivo_	(AUIC)_ex_	E (logCFU/mL)	Calculated PD Target
0	0	0	3.28	E_0_ = 3.28 PD_max_ = 7.58 N = 1.77 ± 0.34 EC_50_ = 67.18 ± 8.32 AUC_24h_/MIC (E = 0) = 57.65 AUC_24h_/MIC (E = −3) = 163.56 AUC_24h_/MIC (E = −4) = 407.12
0.5	0.22 ± 0.05	5.37 ± 1.25	0.31	
1	0.37 ± 0.08	8.96 ± 2.06	−2.31	
2	0.71 ± 0.02	16.83 ± 0.63	−3.63	
4	0.88 ± 0.04	20.28 ± 2.07	−4.30	
6	0.73 ± 0.04	17.93 ± 1.22	−4.30	
8	0.51 ± 0.08	12.18 ± 2.1	−2.93	
12	0.34 ± 0.12	8.12 ± 3.05	−2.21	
24	0.22 ± 0.08	5.27 ± 1.92	−1.47	
36	0.12 ± 0.05	2.77 ± 1.15	1.37	
48	0.07 ± 0.02	1.62 ± 0.49	2.31	
72	0.02 ± 0.009	0.55 ± 0.24	2.82	

## Data Availability

Data is contained within the article.
